# Integrated Analysis of Multiple Microarray Studies to Identify Novel Gene Signatures in Ulcerative Colitis

**DOI:** 10.3389/fgene.2021.697514

**Published:** 2021-07-09

**Authors:** Zi-An Chen, Yu-Feng Sun, Quan-Xu Wang, Hui-Hui Ma, Zhi-Zhao Ma, Chuan-Jie Yang

**Affiliations:** ^1^Department of Gastroenterology, The Second Hospital of Hebei Medical University, Shijiazhuang, China; ^2^Department of Neurosurgery, The Second Hospital of Hebei Medical University, Shijiazhuang, China

**Keywords:** ulcerative colitis, robust rank aggregation, differentially expressed genes, GEO database, microarray

## Abstract

**Background:** Ulcerative colitis (UC) is a chronic, complicated, inflammatory disease with an increasing incidence and prevalence worldwide. However, the intrinsic molecular mechanisms underlying the pathogenesis of UC have not yet been fully elucidated.

**Methods:** All UC datasets published in the GEO database were analyzed and summarized. Subsequently, the robust rank aggregation (RRA) method was used to identify differentially expressed genes (DEGs) between UC patients and controls. Gene functional annotation and PPI network analysis were performed to illustrate the potential functions of the DEGs. Some important functional modules from the protein-protein interaction (PPI) network were identified by molecular complex detection (MCODE), Gene Ontology (GO), and Kyoto Encyclopedia of Genes and Genomes (KEGG), and analyses were performed. The results of CytoHubba, a plug for integrated algorithm for biomolecular interaction networks combined with RRA analysis, were used to identify the hub genes. Finally, a mouse model of UC was established by dextran sulfate sodium salt (DSS) solution to verify the expression of hub genes.

**Results:** A total of 6 datasets met the inclusion criteria (GSE38713, GSE59071, GSE73661, GSE75214, GSE87466, GSE92415). The RRA integrated analysis revealed 208 significant DEGs (132 upregulated genes and 76 downregulated genes). After constructing the PPI network by MCODE plug, modules with the top three scores were listed. The CytoHubba app and RRA identified six hub genes: LCN2, CXCL1, MMP3, IDO1, MMP1, and S100A8. We found through enrichment analysis that these functional modules and hub genes were mainly related to cytokine secretion, immune response, and cancer progression. With the mouse model, we found that the expression of all six hub genes in the UC group was higher than that in the control group (*P* < 0.05).

**Conclusion:** The hub genes analyzed by the RRA method are highly reliable. These findings improve the understanding of the molecular mechanisms in UC pathogenesis.

## Introduction

Ulcerative colitis (UC) is a chronic, complicated, inflammatory disease that affects the colonic mucosa and most commonly presents with abdominal pain, diarrhea, and blood in the stools. Pathological characteristics include relapsing and remitting mucosal inflammation, starting in the rectum and sigmoid colon and extending continuously to proximal segments of the colon or even the entire colon, which leads to permanent fibrosis and tissue damage (Ungaro et al., 2017). The incidence and prevalence of UC have been increasing worldwide. It usually has a long, chronic clinical course, and UC patients are at increased risk of colorectal cancer. Thus, UC has become one of the critical threats and challenges to human health (Kaplan and Ng, 2017; Ng et al., 2017).

At the present, the etiology of UC remains unclear. It is believed that multifactorial pathogenesis plays a role in the occurrence and development of UC, and the factors involved include environmental and psychological factors, dysregulated intestinal flora and immune responses, genetic predisposition, and epithelial barrier defects. The colonic epithelium facilitates host-microorganism interactions to control mucosal immunity, coordinate nutrient recycling and form a mucus barrier. Breakdown of the epithelial barrier is underlying in UC pathogenesis (Ungaro et al., 2017; Parikh et al., 2019). Routinely used indices in the clinical diagnosis and dynamic monitoring of UC include C reactive protein (CRP), erythrocyte sedimentation rate (ESR), and fecal calprotectin, but these lack sensitivity and specificity in differentiating between UC and functional gut disorders (Brookes et al., 2018). Therefore, it is of great importance to further understand the pathogenesis and regulation of UC at the molecular level, as well as the identification of key biomarkers for UC.

In recent years, several studies based on microarray technology have been published to identify effective biomarkers in UC (Cheng et al., 2019, 2020; Chen Y. et al., 2020; Shi et al., 2020; Cao et al., 2021). However, differences in measurement platforms, lab protocols, sample sizes, and operators render gene expression levels incomparable. Based on multiple microarray datasets, robust rank aggregation (RRA) is a method that integrates the results of differential expression analysis, expanding the sample size and reducing the influence of different microarray platforms and imbalanced sample sizes (Kolde et al., 2012). To date, RRA has not been applied in microarray studies of UC. Thus, this study involved a comprehensive evaluation of the published datasets. Based on the inclusion criteria, we screened and included six datasets and identified DEGs using RRA. A protein-protein interaction (PPI) network was constructed to analyze the hub genes, the gene modules, and the involved functions and pathways. Several additional biomarkers were identified that may contribute to the diagnosis of UC, thus providing potential therapeutic targets for patients.

## Materials and Methods

### Search Strategy for the UC Microarray Datasets

**FIGURE 1:**
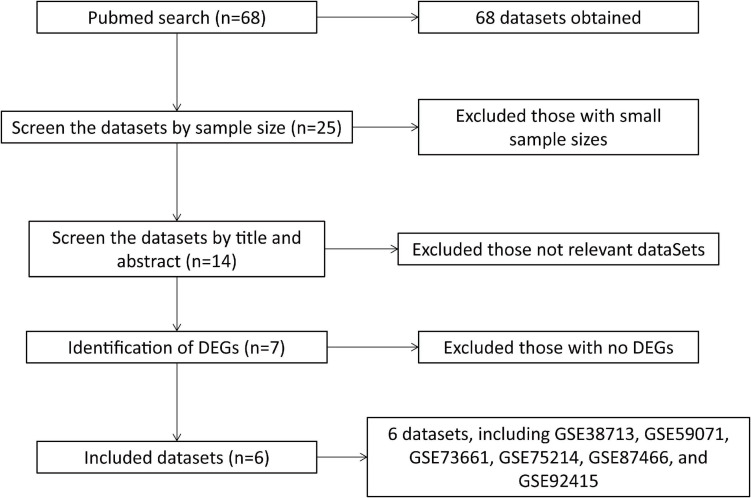
Flowchart of dataset retrieval and inclusion.

A total of 68 datasets were collected from the Gene Expression Omnibus (GEO) Database^1^ by systematic retrieval using the keywords: (“colitis, ulcerative”[MeSH Terms] OR ulcerative colitis [All Fields]) AND “Homo sapiens”[porgn] AND (“Expression profiling by array”[Filter] AND (“2010/01/01”[PDAT]: “2021/03/05”[PDAT])). The inclusion criteria were as follows: (1) a dataset sample size > 30; (2) the dataset included both cases and normal controls; (3) the sample source was “colon,” and (4) the differentially expressed genes (DEGs) with | logFC| > 1.5 and adjusted *P* < 0.05 were identified from the dataset (Figure 1).

### Identification of DEGs in UC

We first downloaded the gene expression profiles from the GEO database for all the datasets included in the final analysis. The scaled expression values for each gene were averaged when multiple probes target the same gene. Second, PERL software was used to extract the matrix file, and the Limma package in R software was used to perform quantile normalization with the normalizeBetweenArrays function. Additionally, we identified the DEGs for each dataset with the criteria of | logFC| > 1.5 and adjusted *P* < 0.05 for comparison with the RRA analysis results.

### RRA Integrated Analysis

In the RRA analysis, DEGs (both upregulated and downregulated) were sorted for each dataset and ranked according to their logFC using the Limma package of R software. Then, all the DEGs were scored according to the ranked list and aggregately analyzed using the RRA package of R software. The adjusted *P*-value in this method reflects the probability of the highly ranked genes in the datasets identified as DEGs. The criteria for the identification of DEGs were set as | logFC| > 1.5 and adjusted *P* < 0.05.

### Functional Annotation

Gene Ontology (GO) is a community-based bioinformatics resource for annotating gene sets, which can be divided into three parts: biological process (BP), cellular component (CC), and molecular function (MF) (Dalmer and Clugston, 2019). The Kyoto Encyclopedia of Genes and Genomes (KEGG) is a database covering various biological signaling pathways. GO enrichment and KEGG pathway analysis provide essential perspectives for bioinformatics analysis. Enrichment analysis for DEGs with the criteria of adjusted *P* < 0.05 was performed using the Cluster Profile package of R software (Yu et al., 2012).

### Protein-Protein Interaction (PPI) Network Analysis

For the obtained DEGs, the String database^2^ was used to construct the PPI network, with the parameter of confidence > 0.4. Visualization of the PPI network was performed by Cytoscape (v3.7.2) (Smoot et al., 2011), and molecular complex detection (MCODE) (a plugin in Cytoscape) (Bader and Hogue, 2003) was used to identify the functional modules. Essential genes were identified by the plugin of CytoHubba (Chin et al., 2014) and sorted by degree scores. The overlap of genes in the PPI network (degree score > 15) and the top 15 DEGs (upregulated or downregulated) in the RRA analysis were determined as hub genes.

### Establishment of UC Model

All animal experiments were performed according to Institutional Animal Care and Use Committee (IACUC) guidelines and were approved by the Ethics Committee at Hebei Medical University.

Sixteen BALB/c mice (male, 6–8 weeks) were obtained from Vital River and were reared in a specific pathogen-free (SPF) environment. All mice were randomly divided into standard control and experimental groups. The mice in the regular control group were given normal drinking water. In contrast, the mice in the experimental group were given a 3.5% DSS solution (dextran sulfate sodium salt, MPbio, MW 36,000–50,000 Da, CA, United States) for 7 days continuously to induce acute colitis and to create the UC animal model. After modeling, all mice were given standard drinking water, kept for another 3 days, and then euthanized with CO_2_. The colon tissue from each mouse was collected. One part was stored in 4% paraformaldehyde (PFA). The other part was homogenized with TRIzol reagent (Invitrogen, Carlsbad, CA, United States), immediately frozen in liquid nitrogen, and stored at −80°C.

### Real-Time Quantitative PCR (RT-qPCR)

Total RNA was extracted according to the manufacturer’s instructions. For examination of mRNA expression, the RNA was reverse transcribed into cDNA, followed by an examination of RT-qPCR using SYBR Mix (CWBIO, Beijing, China). β-actin was indicated as internal controls. All samples were examined in triplicate for each specific gene. The primer sequences for PCR are listed in Supplementary Table 1.

### Pathological Examination

The distal colons of the mice were fixed with 4% PFA and embedded in paraffin. Tissue sections were stained with hematoxylin-eosin (HE). According to the scoring criteria by Dieleman et al. (1998), the intestinal damage level was evaluated under an optical microscope, as shown in Table 1. Three visual fields were randomly selected from each section, and the scores were averaged to determine the damage level of the colonic tissue.

**TABLE 1:** Histological grading of colitis.

Inflammation	Extent	Regeneration	Crypt damage	Percent involvement	Grade
None	None	Complete regeneration or normal tissue	None	None	0
Slight	Mucosa	Almost complete regeneration	Basal 1/3 damaged	1–25%	1
Moderate	Mucosa and submucosa	Regeneration with crypt depletion	Basal 2/3 damaged	26–50%	2
Severe	Transmural	Surface epithelium not intact	Only surface epithelium intact	51–75%	3
		No tissue repair	Entire crypt and epithelium lost	76–100%	4

## Results

### Characteristics of the Included Microarrays

According to the criteria above, a total of six datasets were included in the final analysis: GSE38713 (Planell et al., 2013), GSE59071 (Vanhove et al., 2015), GSE73661 (Arijs et al., 2018), GSE75214 (Vancamelbeke et al., 2017), GSE87466 (Li et al., 2018), and GSE92415. The flowchart of dataset retrieval, inclusion criteria, and exclusion criteria is shown in Figure 1. From the six datasets, a total of 532 cases of UC (including 46 inactive UC cases) were included in the experimental group, and 89 were included in the standard control group. The characteristics of the included microarray datasets are shown in Table 2.

**TABLE 2:** Characteristics of the included microarray datasets.

GSE ID	Participants(control/UC)	Analysis type	Platform	Year	Tissues
GSE38713	13/22	Array	GPL570	2012	Colon
GSE59071	11/97	Array	GPL6244	2015	Colon
GSE73661	12/67	Array	GPL6244	2016	Colon
GSE75214	11/97	Array	GPL6244	2017	Colon
GSE87466	21/87	Array	GPL13158	2018	Colon
GSE92415	21/162	Array	GPL13158	2018	Colon

### Identification of DEGs in UC

**FIGURE 2:**
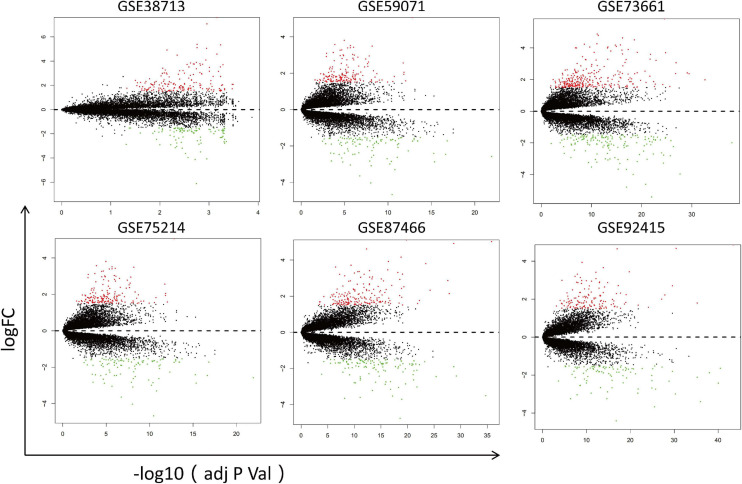
Volcano maps of the six datasets. Red points represent upregulated genes, while green points represent downregulated genes. Black points indicate genes with no significant difference.

First, *the datasets were standardized to correct batch differences* within the datasets (Supplementary Figure 1), showing that the homogeneity of the data met the requirements and could be included in the analysis. Then, the DEGs were identified in each dataset using the Limma package of R software, and the volcano maps are shown in Figure 2.

### RRA Integrated Analysis

**FIGURE 3:**
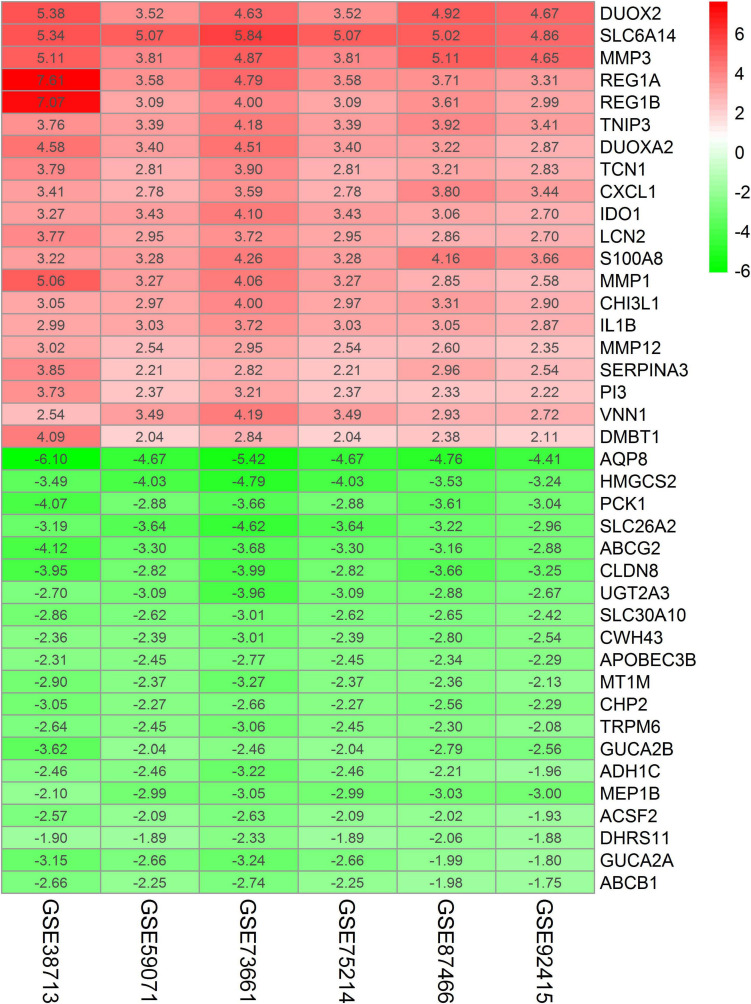
Heatmap of the top 20 DEGs (upregulated or downregulated) identified in the RRA analysis. Red represents a relatively high expression of genes in patients with UC. In contrast, green represents a relatively low expression of genes in patients with UC. The numbers in the heatmap represent logarithmic fold change in each dataset calculated by R software.

A total of 208 DEGs (132 upregulated and 76 downregulated) were identified by RRA analysis, and the heatmap of the top 20 DEGs (upregulated or downregulated) is shown in Figure 3. The top 10 significant genes aberrantly expressed in UC included five upregulated genes [DUOX2 (*P* = 1.66E-18), SLC6A14 (*P* = 1.66E-18), MMP3 (*P* = 6.32E-18), REG1A (*P* = 1.06E-16), REG1B (*P* = 1.95E-15)], and five downregulated genes [AQP8 (*P* = 4.04E-22), HMGCS2 (*P* = 1.89E-17), PCK1 (*P* = 1.06E-16), SLC26A2 (*P* = 2.15E-16), ABCG2 (*P* = 4.04E-16)]. The overall results from RRA analysis are listed in Supplementary Table 2.

Compared with the single dataset analysis, the RRA integrated analysis significantly increased the sample size and reduced the operator influence, thus improving the reliability of the conclusions. In this study, according to the criteria of | logFc| > 1.5 and adjusted *P* < 0.05, 270, 272, 436, 272, 298, and 231 DEGs were identified in each dataset. After excluding duplicates, 666 DEGs were identified, among which 79 common DEGs were identical in all datasets. The DEGs identified in the RRA analysis (*n* = 208) accounted for 40.82–71.69% in each dataset, indicating that RRA integrated the results of the datasets, especially when high-throughput sequencing data were collected from different platforms covering different sets of the gene probes (Table 3).

**TABLE 3:** Comparison of DEGs identified by single dataset analysis and RRA integrated analysis.

GSE ID	RRA analysis	
	Identical DEGs,n (%)	Different DEGs,n (%)
GSE38713	138 (51.11)	132 (48.89)
GSE59071	130 (47.94)	142 (52.21)
GSE73661	178 (40.82)	258 (59.17)
GSE75214	195 (71.69)	77 (28.31)
GSE87466	178 (59.73)	120 (40.27)
GSE92415	156 (67.53)	75 (32.47)
Overall number of unique genes^*a*^	113 (16.97)	553 (83.03)
Common genes with significant difference^*b*^	79 (100)	0 (0)

*^*a*^The genes that all differentially expressed genes identified by six datasets.*

*^*b*^The genes that were commonly identified by six datasets.*

### Functional Annotation

**FIGURE 4:**
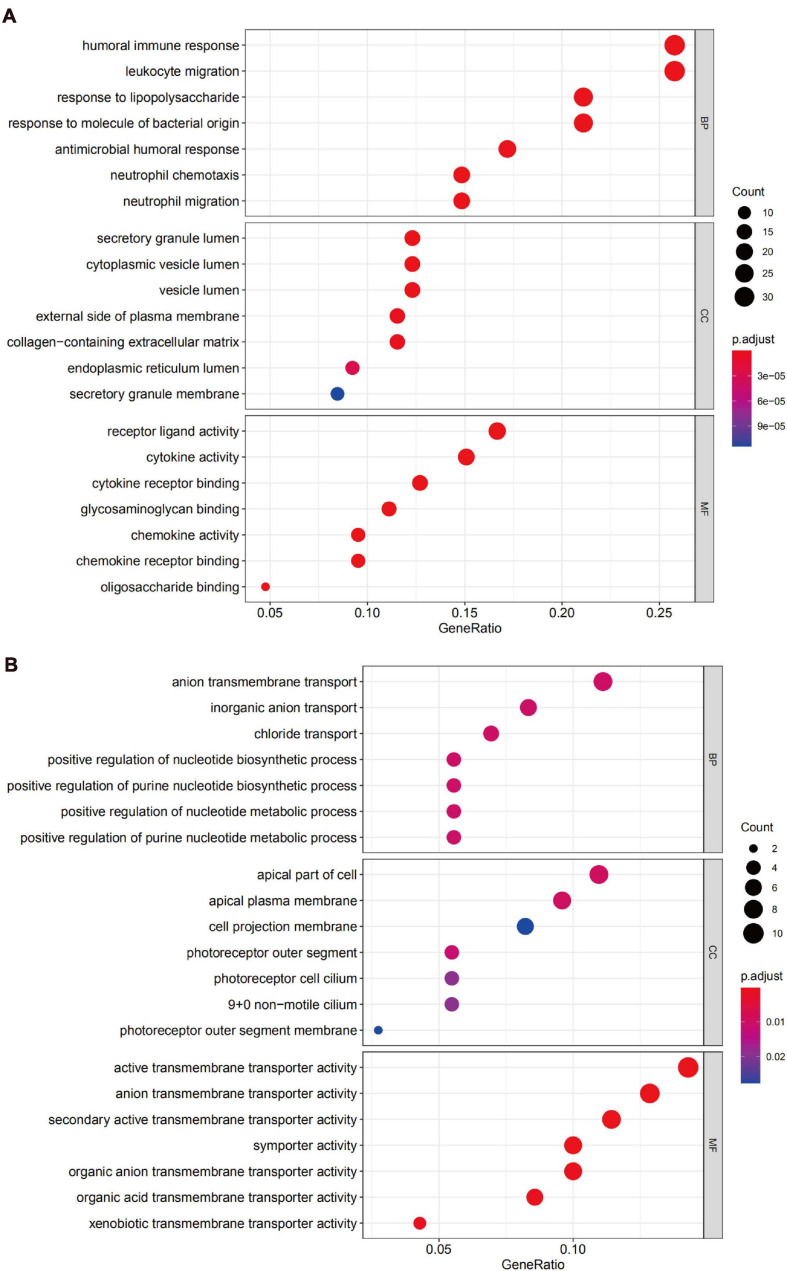
Gene Ontology (GO) analysis of DEGs. **(A)** Functional enrichment analysis of upregulated genes. **(B)** Functional enrichment analysis of downregulated genes.

We uploaded the 132 upregulated and 76 downregulated DEGs to perform GO (including biological process, molecular function, and cellular component) analysis and KEGG analysis. The results indicated that the upregulated DEGs were particularly enriched in humoral immune response, in leukocyte migration, in response to lipopolysaccharide, in the secretory granule lumen, in the cytoplasmic vesicle lumen, in the vesicle lumen, in receptor-ligand activity, in cytokine activity, and cytokine receptor binding and were the top three enriched terms, depending on the *P*-value of the respective categories (Figure 4A). The downregulated DEGs were mainly enriched in anion transmembrane transport and inorganic anion transport based on the *P*-value (Figure 4B).

**FIGURE 5:**
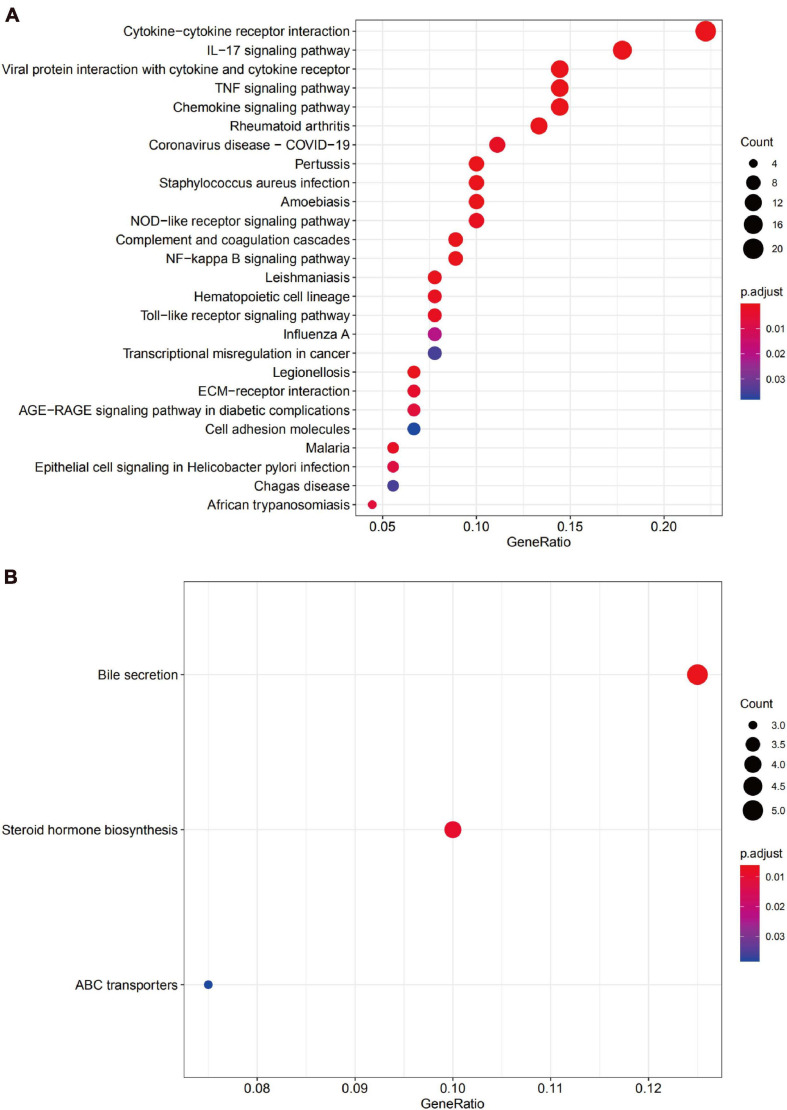
The Kyoto Encyclopedia of Gene and Genome (KEGG) pathway enrichment analysis for DEGs. **(A)** The functional enrichment analysis of upregulated genes. **(B)** Functional enrichment analysis of downregulated genes.

The KEGG pathway enrichment analysis suggested that the upregulated DEGs predominantly participated in inflammation-related pathways, including the cytokine-cytokine receptor interaction and IL-17 signaling pathway, while the downregulated genes were mainly enriched in the bile secretion pathway (Figures 5A,B).

### PPI Network Analysis and Identification of Hub Genes

**FIGURE 6:**
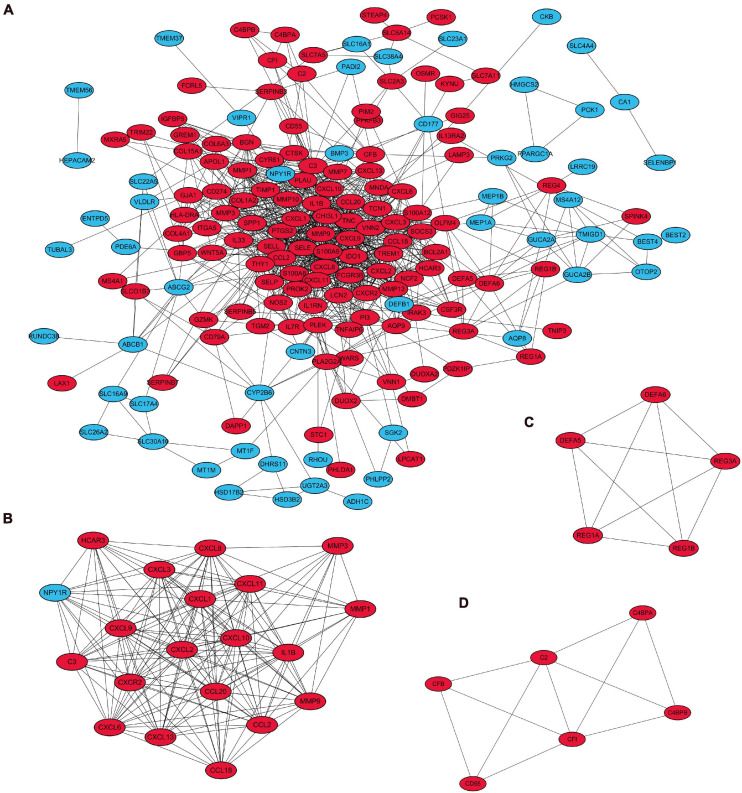
Visualization and module identification of the PPI network. **(A)** A total of 208 DEGs were mapped using Cytoscape software. Three modules of the PPI networks were identified by the MCODE plug-in. **(B)** Module 1 comprised CXCL6, CXCL8, CCL18, C3, HCAR3, CXCL, CXCL13, IL1β, NPY1R, MMP3, CCL20, MMP1, CXCR2, CCL2, CXCL1, CXCL10, CXCL9, CXCL3, and CXCL11 with the seed gene MMP9. **(C)** Module 2 contained DEFA5, DEFA6, REG3A, and REG1B with the seed gene REG1A, and **(D)** module 3 consisted of C2, C4BPA, CFI, CFB, and CD55 with the seed gene C4BPB. The red points represent upregulated genes, while the blue points represent downregulated genes.

A visual network of DEGs identified from the RRA analysis was constructed using the String (Search Tool for the Retrieval of Interacting Genes database) website, and these were comprised of 205 nodes and 880 edges. The network was then imported into Cytoscape for subsequent genetic analysis (Figure 6A).

The top three modules with the highest scores were identified by MCODE (Figures 6B–D). Module 1 comprised CXCL6, CXCL8, CCL18, C3, HCAR3, CXCL, CXCL13, IL1β, NPY1R, MMP3, CCL20, MMP1, CXCR2, CCL2, CXCL1, CXCL10, CXCL9, CXCL3, and CXCL11 with the seed gene MMP9; module 2 contained DEFA5, DEFA6, REG3A, and REG1B with the seed gene REG1A; and module 3 consisted of C2, C4BPA, CFI, CFB, and CD55 with the seed gene C4BPB. The score of each module is shown in Supplementary Table 3.

**FIGURE 7:**
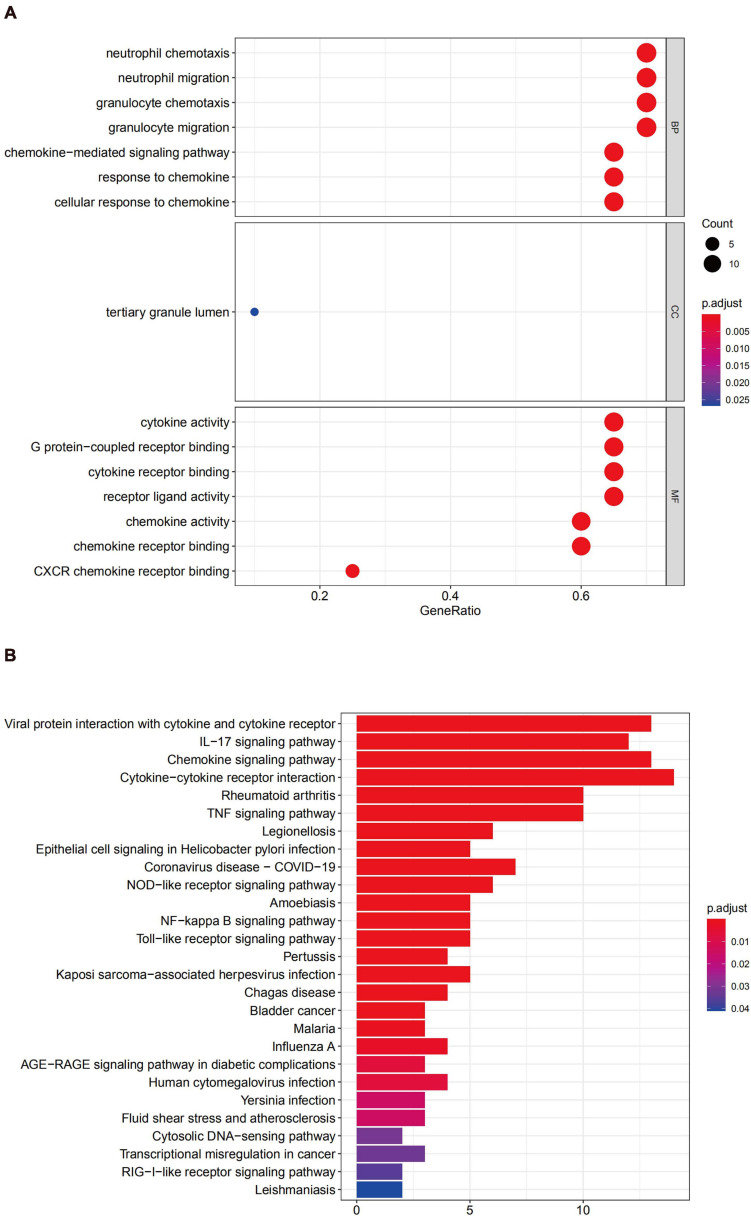
Functional enrichment analysis for the genes in module 1 **(A)** GO analysis for DEGs. **(B)** The KEGG analysis for DEGs.

GO enrichment analysis of module 1 showed that the genes were mainly related to inflammatory cell migration and cytokine activity (Figure 7A), and KEGG analysis revealed that these genes were mainly involved in cytokine-cytokine receptor interactions, viral protein interactions with cytokines and cytokine receptors, chemokine signaling pathways, and IL-17 signaling pathways (Figure 7B).

**FIGURE 8:**
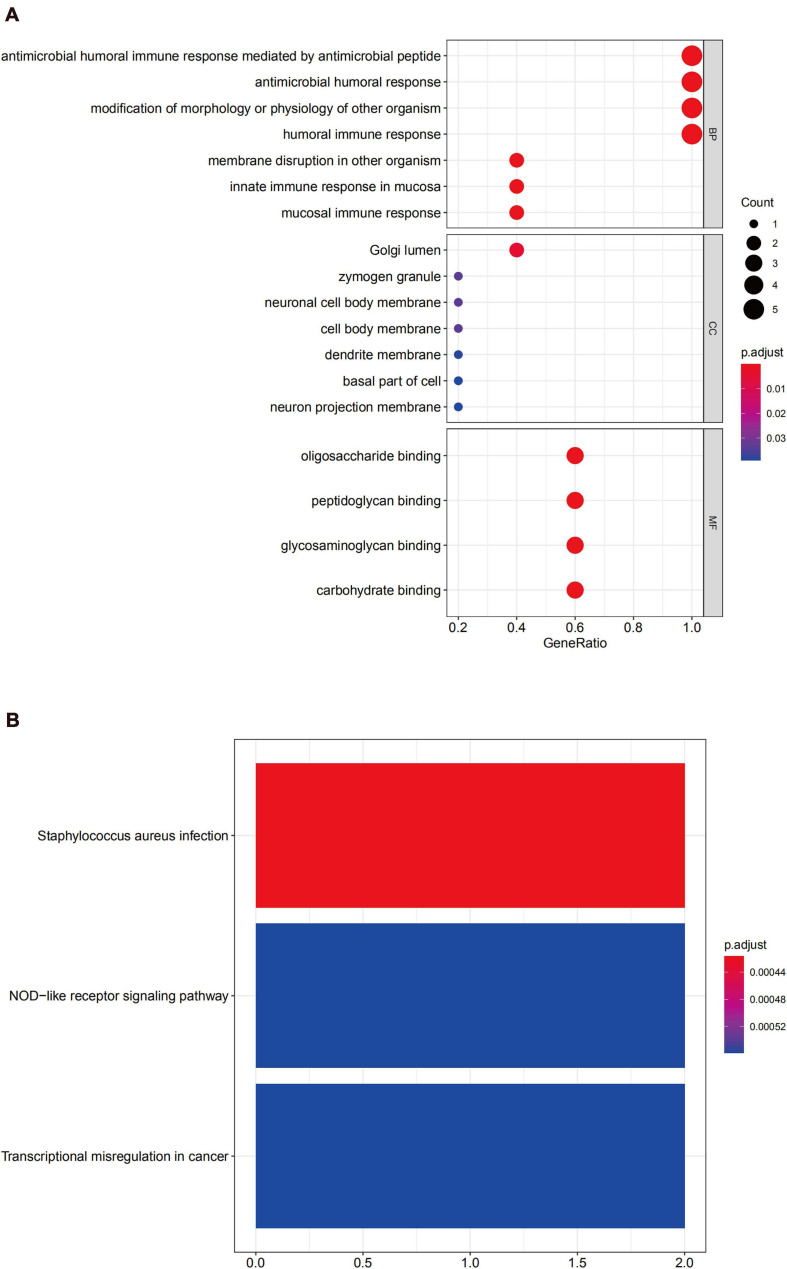
Functional enrichment analysis for the genes in module 2 **(A)** GO analysis for DEGs. **(B)** The KEGG analysis for DEGs.

GO enrichment analysis of module 2 revealed that the DEGs were mainly related to the humoral immune response (Figure 8A). The KEGG analysis revealed that these genes were mainly involved with Staphylococcus aureus infection, in the NOD-like receptor signaling pathway, and transcriptional misregulation in cancer (Figure 8B).

**FIGURE 9:**
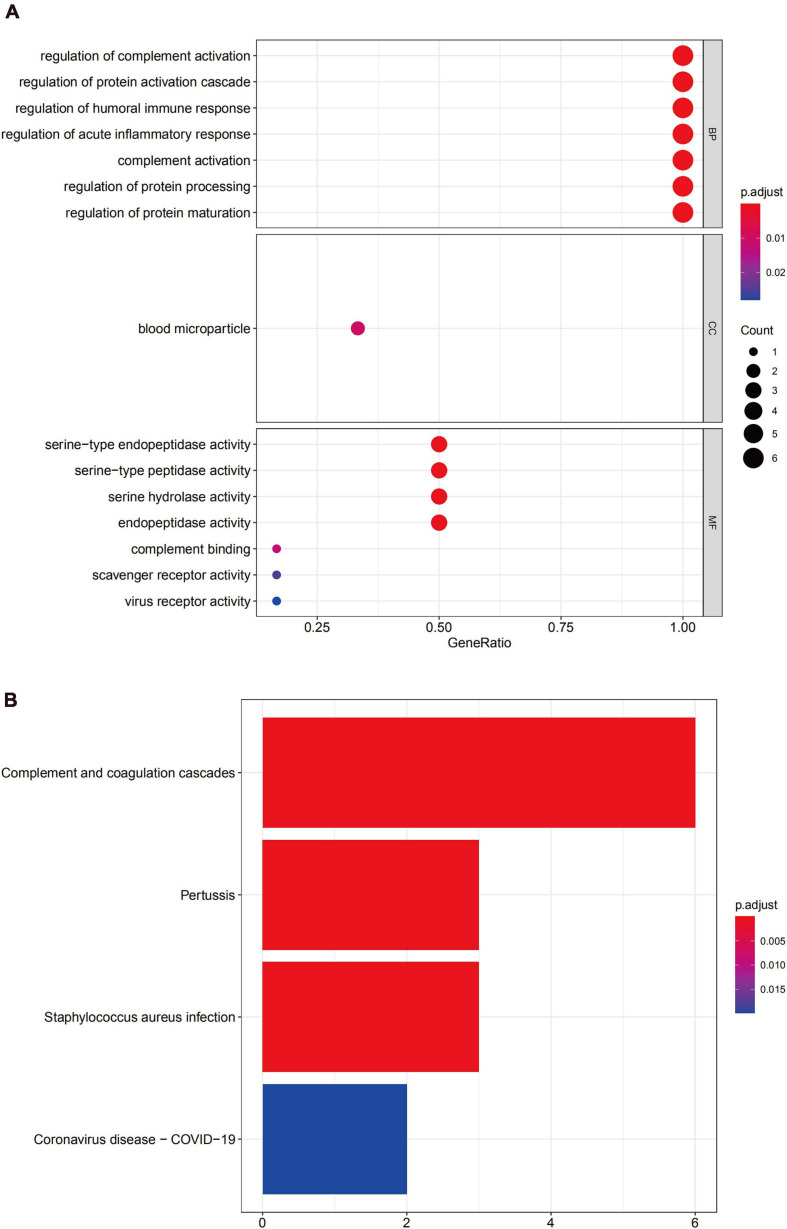
Functional enrichment analysis for the genes in module 3 **(A)** GO analysis for DEGs. **(B)** The KEGG analysis for DEGs.

GO enrichment analysis of module 3 suggested that the DEGs were mainly related to the regulation of complement activation, to the regulation of the protein activation cascade, and the regulation of the humoral immune response (Figure 9A), and KEGG analysis revealed that these genes were mainly involved in complement and the coagulation cascade-related pathways (Figure 9B).

### The Determination of Hub Genes

CytoHubba was used to identify the critical genes in the PPI network and sort them by degree scores. Integrating the RRA analysis results, six hub genes were obtained, including LCN2, CXCL1, MMP3, IDO1, MMP1, and S100A8. All results are listed in Supplementary Table 4.

### Verification of Hub Genes in a Mouse UC Model

**FIGURE 10:**
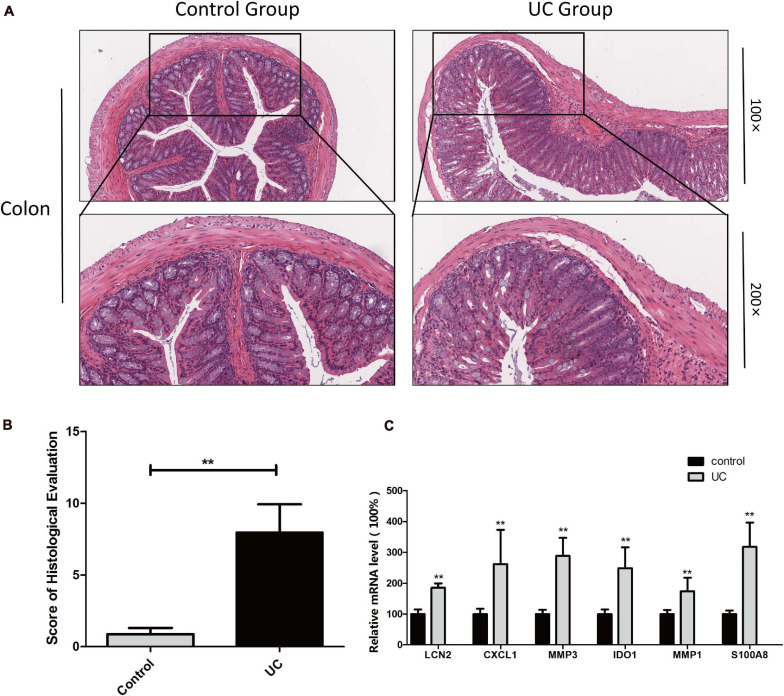
Determination of hub genes in mouse UC model H&E staining of colon tissues from the control and dextran sulfate sodium-induced colitis model mice **(A)** and histological lesion score of colon tissues **(B)** were performed. The expression of hub genes was examined by qPCR, and β-actin served as an internal reference **(C)**. **Indicates *p* < 0.05.

Finally, the expression of the six hub genes was verified in a mouse UC model. All mice were alive at the end of the experiment (*n* = 8). UC was confirmed by the pathological examination of the colonic tissue. The normal structure of the colonic tissue in UC mice was almost gone, with inflammatory cell infiltration into the submucosa. Hence, the pathological score of the experimental group was significantly higher than that of the normal control group (Figures 10A,B).

The expression of the six hub genes in colon tissue was quantified by qPCR, showing that the expression was significantly higher in the experimental group than in the normal control group (Figure 10C).

## Discussion

UC is an intestinal inflammation disease with multiple causes; it is becoming increasingly common and is characterized by prolonged clinical courses and recurrent attacks. The etiology remains unclear, as the genetic, environmental, and psychological factors involved in the pathogenesis make interpreting its pathological mechanism and diagnosis difficult (Kaplan and Ng, 2017; Ungaro et al., 2017). In this study, several published datasets were combined for bioinformatics analysis.

GEO (see text footnote 1) is an international public repository for high-throughput microarray and next-generation sequence functional genomic datasets submitted by the research community. Currently, it is the world’s largest public database for storing gene expression data, so it was searched to identify relevant UC datasets. In total, six datasets were retrieved and combined for RRA analysis, which identified 208 DEGs. There are several methods for the combination of multiple microarrays to perform bioinformatics analysis. Batch normalization can integrate different datasets. However, differences in measurement platforms, lab protocols, sample sizes, and operators render gene expression levels incomparable. In recent years, several studies based on microarray technology have been published to identify effective biomarkers in UC, most of which are prone to utilizing intersecting genes from different microarrays to perform analyses (Cheng et al., 2019, 2020; Chen Y. et al., 2020; Shi et al., 2020; Cao et al., 2021). As shown in Table 3, these methods can be applied to fewer datasets (≤3 datasets) because more datasets represent overly strict inclusion criteria, leading to fewer DEGs. In addition, the results based on intersecting genes are prone to be influenced by a single abnormal dataset. In contrast, RRA analysis focuses on the ranking of each gene in each dataset. With the assumption that each gene identified in each dataset is randomly arranged, the RRA compares the ranking of a randomly ordered list with the baseline case, and a higher gene rank is associated with a lower *P*-value. To our knowledge, RRA was first used to integrate datasets in UC and has preliminarily proven its reliability via our investigation in animal models. Notably, there are dozens of ways to perform meta-analyses of different studies, and RRA is only one of them. We reviewed the current studies on ulcerative colitis (Bopanna et al., 2017; Gubatan et al., 2019; Szemes et al., 2019; Chen M. et al., 2020; Li et al., 2020; Sankarasubramanian et al., 2020; Yoon et al., 2020; de Carvalho et al., 2021; Ye et al., 2021). We believe that the advantage of RRA analysis is that more potential biomarkers can be found at one time, which can provide clues for subsequent research. At the same time, the data were collected from microarray analysis to avoid the interference of human factors such as improper blind method. However, there are also some shortcomings, such as the biomarkers may come from data overfitting, and the follow-up validation of these biomarkers still needs follow-up experimental studies, clinical validation, and multi-center clinical trials.

PPI network analysis was performed for all DEGs. MCODE, an algorithm that allows the automated prediction of protein complexes from qualitative protein-protein interaction data, was used to identify multiple functional gene modules (Bader and Hogue, 2003). The top three modules with the highest scores were further analyzed, revealing that module 1 was mainly related to inflammation, with the genes involved in the chemotaxis, aggregation, and cytokine activity of inflammatory cells, and these genes in module 1 were previously reported on in several bioinformatic analyses of UC. The genes in modules 2 and 3 have been less frequently reported on in the literature; however, all genes were upregulated in UC. Module 2 comprised REG1A, DEFA5, DEFA6, REG3A, and REG1B. The results from the GO analysis linked module 2 to various humoral responses, while KEGG analysis showed that it was primarily involved in Staphylococcus aureus infection. Studies have reported that the severity of UC is related to the imbalanced intestinal flora in patients. Intestinal antigens from intestinal bacteria and their metabolites are common, with various antibodies produced in the intestinal immune response (Frehn et al., 2014; Jansen et al., 2016; Soontararak et al., 2019). Additionally, some studies have shown that the REG family proteins play a role in mucosal regeneration in UC (Sekikawa et al., 2010; Tsuchida et al., 2017; Takasawa et al., 2018). Module 3 contained C4BPB and was mainly related to the regulation of complement activation, while KEGG analysis revealed that it mainly participated in complement and coagulation cascades. The complement system is part of the innate sensor and effector systems, such as the Toll-like receptors (TLRs), which recognize and quickly systemically and locally respond to microbial-associated molecular patterns (MAMPs) with a tailored defense reaction. MAMP recognition of microbial-associated molecular patterns by intestinal epithelial cells (IECs) and appropriate immune responses are of significant importance for maintaining intestinal barrier function. Proper activation of the intestinal complement system might play an essential role in resolving chronic intestinal inflammation, while overactivation and/or dysregulation might worsen intestinal inflammation. Hence, how IECs, intestinal bacteria, and epithelial cells express complement and interact in the long-term course of UC remains to be elucidated (Geremia et al., 2014; Sina et al., 2018). Furthermore, the activation of the complement system may promote UC-associated carcinogenesis (Ning et al., 2015).

The DEGs were screened using CytoHubba, a novel Cytoscape plugin for scoring and ranking nodes in a network through different algorithms to evaluate the importance of nodes and gene connectivity in a biological network. It provides eleven topological analysis methods, among which degree is the most commonly used (Chin et al., 2014). The integrated analysis of the top 20 DEGs ranked by degree scores (≥ 15), and RRA identified six potential hub genes, LCN2, CXCL1, MMP3, IDO1, MMP1, and S100A8.

The C-X-C motif chemokine ligand 1 (CXCL1) has been implicated in the malignant behavior of solid and hematological neoplasms in combination with the C-X-C motif chemokine receptor 2 (CXCR2), and these two ligands act indirectly on tumor angiogenesis by regulating the trafficking of leukocytes that produce angiogenic factors and a variety of inflammatory cytokines (Mantovani et al., 2010). Recently, studies have shown that the CXCL1/CXCR2 signaling pathway could regulate the inflammatory response and promote tumor cell proliferation, invasion, and transvascular metastasis, acting as essential molecules in the progression of inflammation (Acharyya et al., 2012). Studies have also shown that the blockage of CXCR2 in neutrophils by a selective inhibitor could significantly alleviate the symptoms of DSS-induced colitis in mice and could suppress the production of proinflammatory cytokines. Hence, CXCR2 is a potential target for UC treatment (Zhu et al., 2020). LCN2 (Østvik et al., 2013; Stallhofer et al., 2015; Buisson et al., 2018; Zollner et al., 2021) and S100A8 (Manolakis et al., 2011; Azramezani Kopi et al., 2019; Okada et al., 2019, 2020) are critical proinflammatory cytokines that have been reported in recent studies as potential molecular markers of UC in serum and stool samples. LCN2 and S100A8 also have antimicrobial effects and may be involved in the regulation of intestinal flora as antimicrobial peptides, which may be indirectly related to UC. Host and microbial tryptophan (Trp) metabolism have emerged as critical regulators in mucosal homeostasis. Indoleamine 2,3 dioxygenase-1 (IDO1) is the first enzyme in Trp metabolism in the kynurenine (Kyn) pathway and is perhaps most relevant in the context of homeostasis. Therefore, IDO1 may be an important molecular marker (Sofia et al., 2018; Alvarado et al., 2019). Although the specific mechanisms of IDO1 remain obscure, numerous studies have shown an increased expression of IDO1 in inflammatory bowel disease, infection, and diverticulosis (Ferdinande et al., 2008; Nikolaus et al., 2017; Vancamelbeke et al., 2017). IDO1 has also been closely related to disease remission, with genetic abnormalities or drug inhibition of IDO1 aggravating the disease (Ciorba et al., 2010; Gupta et al., 2012; Nikolaus et al., 2017).

In recent years, the association between UC and colon cancer has attracted significant attention. CXCL1, LCN2, S100A8, and IDO1 may play essential roles in cancer progression. Studies have shown that IDO1 could promote colitis-associated tumorigenesis in mice. This approach revealed a cell-autonomous mechanism by which IDO1 tryptophan catabolites (kynurenine and quinolinic acid) directly promote cancer cell proliferation (Thaker et al., 2013). Further research is needed regarding the related evolutionary mechanism of UC and colon cancer.

Matrix metalloproteinases (MMPs) belong to a family of zinc-dependent endopeptidases, which are mainly produced and secreted by connective tissue, endothelial cells, mononuclear macrophages, neutrophils, and tumor cells. MMPs participate in the degradation of ECM components (Karamanos et al., 2021), with increased expression in the UC lesion area (Schuppan and Hahn, 2000; Medina et al., 2003; Wang and Yan, 2006; Meijer et al., 2007). Additionally, genetic variations in MMPs may be associated with an increased risk of UC differences in clinical symptoms (Morgan et al., 2011). Studies have shown that the overexpression of MMP-1 and MMP-3 play an important role in the pathogenesis of steroid-dependent uncreative colitis (SDUC). The protein expressions of MMP-1 and MMP-3 significantly increased in the healing regions of colonic tissues in SDUC remission patients but not in non-SDUC remission patients, suggesting that the overexpression of MMP-1 and MMP-3 are not the only factors involved in the pathogenesis of UC but also are a critical feature in the steroid dependency in UC (Wang and Qiu, 2010).

The expression of the six hub genes was confirmed in the DSS-induced UC mouse model. In published studies, some scholars screened diagnostic biomarkers for UC from the DEGs identified from a single dataset or an overlap of two or three datasets via bioinformatics analyses (Cheng et al., 2019, 2020; Chen Y. et al., 2020; Shi et al., 2020; Cao et al., 2021). In contrast, the advantage of this research lies in the larger dataset obtained by combining data from six GEO datasets, which increased the sample size and ensured the stability and relative reliability of the conclusions. On the other hand, the RRA method was used to reduce the influences of the measurement platform, the sample size of datasets, the experimental design, and other factors on the final results.

## Conclusion

In conclusion, six datasets were integrated for bioinformatics analyses and identified three functional gene modules and six hub genes, the expression of which was confirmed in a mouse model of UC. These results will help to further explore the mechanisms related to the occurrence and development of UC and to provide potential targets for the detection and treatment of UC patients in the future.

## Data Availability Statement

The original contributions presented in the study are included in the article/Supplementary Material, further inquiries can be directed to the corresponding author/s.

## Ethics Statement

The animal study was reviewed and approved by The Ethics Committee at the Hebei Medical University.

## Author Contributions

Z-AC collected the manuscripts and analyzed the data, analyzed the conclusions, and drafted the manuscript. Y-FS reviewed the data and conclusions. Q-XW and H-HM contributed to writing. C-JY and Z-ZM presented the idea of this manuscript, supported the funding, analyzed the conclusions, and drafted and revised the manuscript. All authors contributed to the article and approved the submitted version.

## Conflict of Interest

The authors declare that the research was conducted in the absence of any commercial or financial relationships that could be construed as a potential conflict of interest.

## Footnotes

1 https://www.ncbi.nlm.nih.gov/geo/

2 https://www.string-db.org/
