# Supplementary material for: Integrated Analysis of Multiple Microarray Studies to Identify Novel Gene Signatures in Ulcerative Colitis
Source: Front Genet. 2021 Jul 9;12:697514. doi: 10.3389/fgene.2021.697514 (PMC8299473; doi:10.3389/fgene.2021.697514)
Supplement: Supplementary file 1 [file Table_1.DOCX]

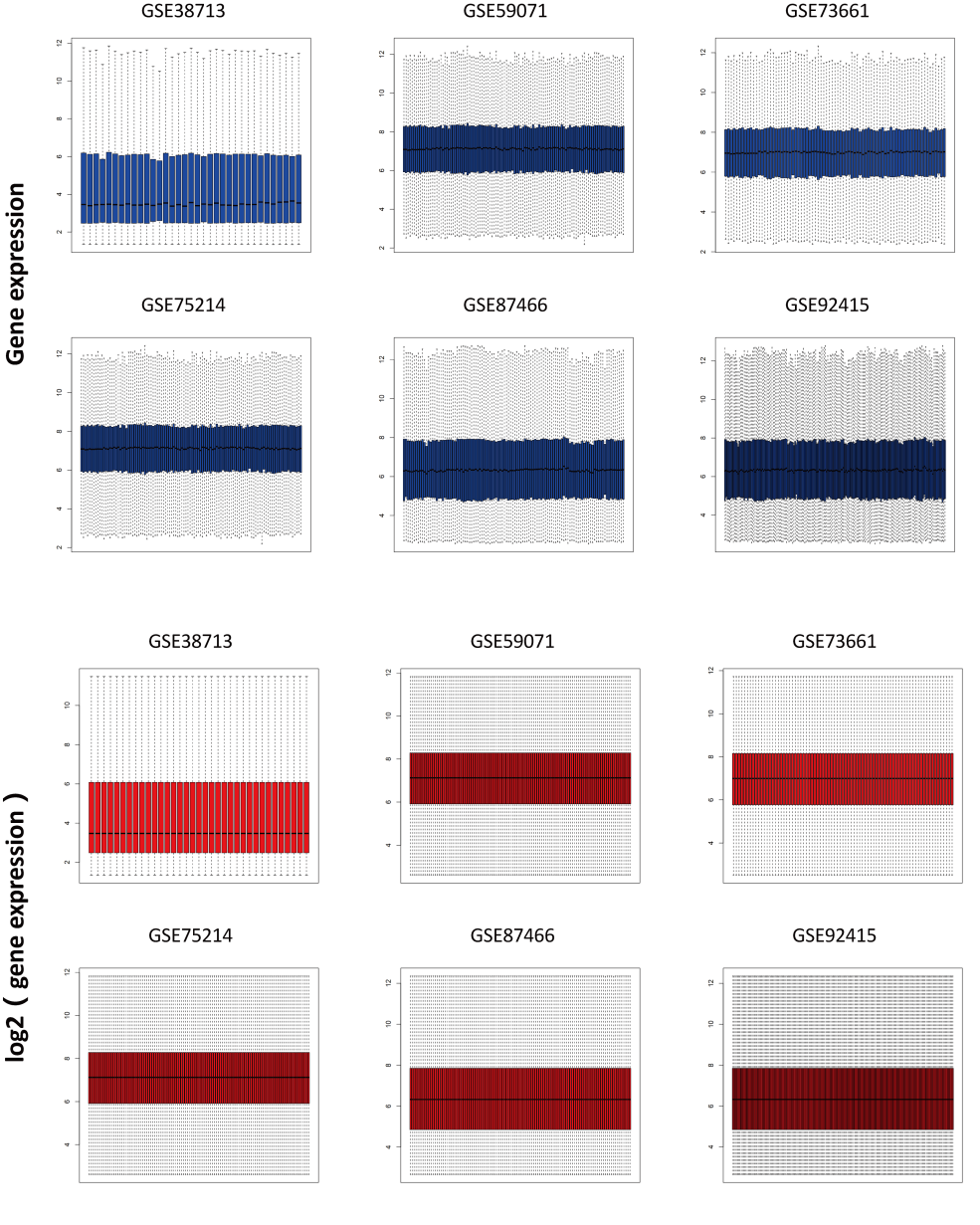


**Supplementary Figure 1：Standardization of gene expression**. Standardization of gene expression in GSE387713, GSE59071, GSE73661, GSE75214, GSE87466, GSE92415 datasets. The blue bar represents the data before normalization, and the red bar represents the data after normalization.
